# Serotyping of *Streptococcus pneumoniae* Based on Capsular Genes Polymorphisms

**DOI:** 10.1371/journal.pone.0076197

**Published:** 2013-09-24

**Authors:** Frédéric Raymond, Nancy Boucher, Robin Allary, Lynda Robitaille, Brigitte Lefebvre, Cécile Tremblay, Jacques Corbeil, Alain Gervaix

**Affiliations:** 1 Centre de Recherche en Infectiologie and Département de Microbiologie et Immunologie, Faculté de Médecine, Université Laval, Québec, Québec, Canada; 2 Laboratoire de Santé Publique du Québec, Institut National de Santé Publique, Ste-Anne-de-Bellevue, Québec, Canada; 3 Department of Pediatrics, University Hospitals of Geneva, Geneva, Switzerland; Rockefeller University, United States of America

## Abstract

*Streptococcus pneumoniae* serotype epidemiology is essential since serotype replacement is a concern when introducing new polysaccharide-conjugate vaccines. A novel PCR-based automated microarray assay was developed to assist in the tracking of the serotypes. Autolysin, pneumolysin and eight genes located in the capsular operon were amplified using multiplex PCR. This step was followed by a tagged fluorescent primer extension step targeting serotype-specific polymorphisms. The tagged primers were then hybridized to a microarray. Results were exported to an expert system to identify capsular serotypes. The assay was validated on 166 cultured *S. pneumoniae* samples from 63 different serotypes as determined by the Quellung method. We show that typing only 12 polymorphisms located in the capsular operon allows the identification at the serotype level of 22 serotypes and the assignation of 24 other serotypes to a subgroup of serotypes. Overall, 126 samples (75.9%) were correctly serotyped, 14 were assigned to a member of the same serogroup, 8 rare serotypes were erroneously serotyped, and 18 gave negative serotyping results. Most of the discrepancies involved rare serotypes or serotypes that are difficult to discriminate using a DNA-based approach, for example 6A and 6B. The assay was also tested on clinical specimens including 43 cerebrospinal fluid samples from patients with meningitis and 59 nasopharyngeal aspirates from bacterial pneumonia patients. Overall, 89% of specimens positive for pneumolysin were serotyped, demonstrating that this method does not require culture to serotype clinical specimens. The assay showed no cross-reactivity for 24 relevant bacterial species found in these types of samples. The limit of detection for serotyping and *S. pneumoniae* detection was 100 genome equivalent per reaction. This automated assay is amenable to clinical testing and does not require any culturing of the samples. The assay will be useful for the evaluation of serotype prevalence changes after new conjugate vaccines introduction.

## Introduction

*Streptococcus pneumoniae* is a major pathogen causing a wide spectrum of infections ranging from acute otitis media and lower respiratory tract infection to severe invasive diseases, such as septicemia and meningitis. Recent estimates of childhood mortality due to *S. pneumoniae* range from 700,000 to 1 million deaths yearly worldwide [1].

Among the >92 capsular serotypes of *S. pneumoniae* identified thus far, seven (4, 6B, 9V, 14, 18C, 19F and 23F) were responsible for 65 to 85% of invasive pneumococcal diseases (IPD) in children worldwide in the year 2000 [2]. A conjugate pneumococcal vaccine containing these serotypes (PCV7, Prevenar®, Pfizer) was licensed in 2000. Introduction of this vaccine rapidly led to a dramatic decrease in the incidence of IPD from an average of 25.1 cases/100,000 persons in 1999 to 12.6 cases/100,000 persons in 2004 in the USA [3]. Large post-licensure vaccine surveillance studies have raised concerns about an increased incidence of invasive disease due to non-vaccine serotypes, especially serotypes 3, 7F, 19A, 22 and 33 [4,5], a phenomenon known as serotype replacement. A 13-valent pneumococcal vaccine including the PCV7 serotypes as well as serotypes 1, 3, 5, 6A, 7F and 19A was licensed in 2010 (PCV13, Prevnar 13®, Pfizer). This new vaccine and others (Synflorix®, GSK) have the potential to further decrease IPD rates and prevent resurgence of pre-PCV7 IPD rates. However, ongoing serotype replacement is still of concern. Consequently, serotyping strains remains of paramount importance to both assess the effectiveness of current vaccines and to closely monitor the emergence of non-vaccine strains.

*S. pneumoniae* serotyping was developed at the beginning of the 20th century using panels of specific anti-sera produced in animals and directed against polysaccharides of the pneumococcal capsule. These agglutination assays are complex, costly and require highly skilled personnel [6]. Traditional agglutination assays cannot be performed directly on clinical samples since growth of *S. pneumoniae* on culture media is required. However, culture is often negative if patients received antibiotics before sampling of blood, cerebrospinal fluid (CSF) or other biological fluids, and in children with pneumococcal pneumonia in whom blood culture tends to be negative but PCR-positive for *S. pneumoniae* [7,8]. In recent years, immunological assays based on ELISA or latex-bead agglutination have been shown to work directly on clinical specimens [9–11]. For example, Sheppard and collaborators show that a multiplex immunoassay using xMAP beads can identify 14 *S. pneumoniae* serotypes directly from CSF [12].

Genes coding for the bacterial capsule are part of a complex gene structure where multiple and subtle polymorphisms in the capsular operon result in different serotypes. PCR-based serotyping using primers that amplify serotype-specific sequences are considered the method of choice for non-cultivable pneumococci serotyping [13,14]. A sequential multiplex PCR approach was initially proposed by Pai and collaborators [15] to identify 29 serotypes and has later been optimized for geographical locations such as South-Saharan Africa [16], Latin America [17] and Bangladesh [18]. Siira and collaborators complemented sequential multiplex PCR reactions by Quellung testing to increase confidence for 13 serogroups of the 42 serotypes/serogroups detected by PCR [6]. Selva and collaborators proposed a method that uses 3 multiplex PCR followed by fragment analysis and automated capillary electrophoresis to identify 40 serotypes or groups of serotypes [19]. Coskun-Ari and collaborators published a single multiplex that allows the identification of the 13 serotypes included in PCV13 [20]. Ahn and collaborators were able to identify 35 serotypes using as series of 8 multiplex PCR reactions [21]. Pimenta and collaborators described a series of multiplex real-time PCR that allowed the identification of 21 serogroups or serotypes, the order of which can be optimized for different regions [22]. PCR-based serotyping of *S. pneumoniae* has also been coupled with reverse line blot hybridization [23], restriction fragment length polymorphism [24,25] or amplicon sequencing [26,27]. This latter method, also termed sequetyping, relies on the sequencing of capsular operon genes such as *cpsB* in order to serotype samples by comparing the sequence obtained to those published by Bentley and collaborators [28]. Although these methods are cost-effective and specific, some serotypes are difficult to discriminate. As an example, a single nucleotide polymorphism in the *wciP* gene results in two distinct capsular serotypes, namely 6A and 6B, emphasizing the inability of PCR-based methods to realistically be used to differentiate serotypes from closely related serogroups [29]. Methods using PCR fragment length [30] or pyrosequencing [29] have been proposed to solve this problem, but are specific to serogroup 6. Immunological methods remain the most reliable approaches to discriminate closely related serotypes such as 6A and 6B. Microarray-based methods have also been published. Scott and coworkers used a microarray targeting capsular operon genes to serotype pneumococcal isolates [31]. Tomita and collaborators used a targeted microarray to identify 23 serotypes [32]. Both studies used conventional glass microarray supports, which require highly skilled personnel and are not amenable to clinical laboratories or to conduct large studies.

**Figure 1:**
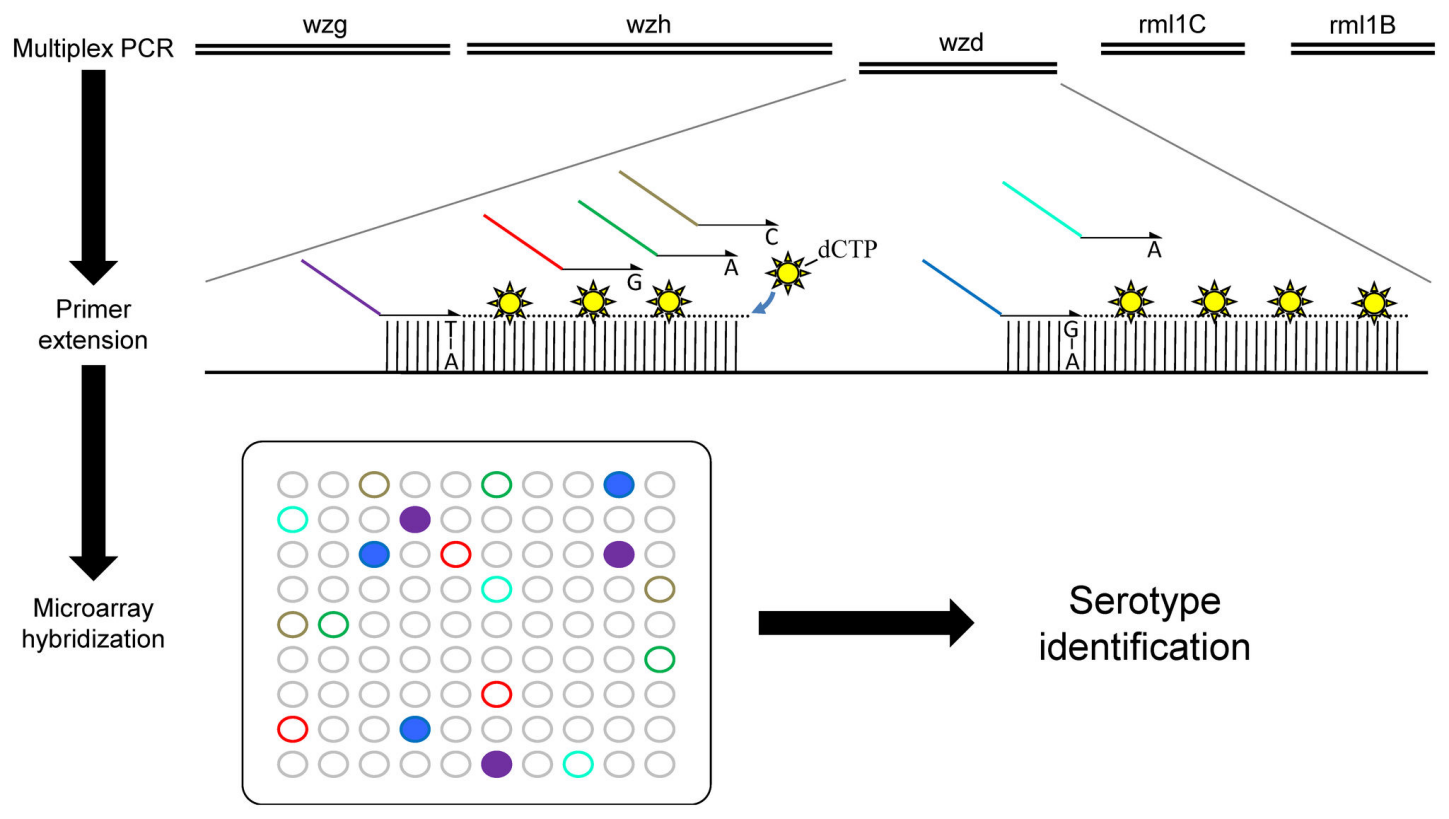
Schematic representation of experimental procedure. Multiplex PCR amplifies up to 12 genes and is performed outside of the INFINITI system. Amplification products are loaded in the INFINITI system at the primer extension step. Primer extension oligonucleotides include a tag sequence that will hybridize to the microarray and a specific detection sequence that allows for primer extension of *S. pneumoniae* genes. Fluorescent nucleotides are incorporated during primer extension. Once the reaction is complete, the INFINITI system automatically transfers the labeled products to the microarray for hybridization. Tag sequences hybridize to anti-tags located on the microarray. Microarrays are then washed, dried and loaded into the integrated confocal scanner where fluorescence is measured. The report generated by the instruments is analyzed off-line using the Pneumotyper software.

**Figure 2:**
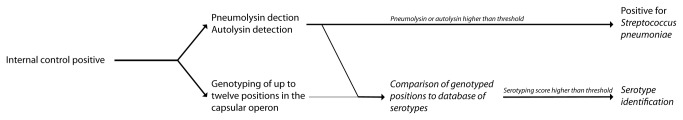
Algorithm for results analysis. Samples were assessed for the presence of *S. pneumoniae* by detecting the pneumolysin and the autolysin genes. If *S. pneumoniae* was detected in the sample, the serotyping probes were analyzed in order to identify the serotype.

Immunological and molecular serotyping methods often include serotypes based on their prevalence, an approach that can bias serotyping studies. Moreover, most DNA-based methods allow the identification of a limited number of serotypes or serogroups. Herein, we suggest an innovative serotyping approach that, instead of detecting one target per serotype, relies on the genotyping of 12 positions located in the capsular operon to identify pneumococcal serotypes. This assay constitutes a proof-of-concept that typing a limited number of polymorphisms could allow a fast and efficient serotyping of *S. pneumoniae*. To facilitate large-scale clinical studies, the assay was adapted for the AutoGenomics INFINITI system and it could be readily ported to another automated platform. A flowchart exemplifying the steps of the assay and data analysis are shown in Figure 1 and Figure 2, respectively.

## Methods

### Strains and clinical samples

One hundred and sixty-six (166) isolates of *S. pneumoniae*, which had been previously serotyped using the Quellung method, were provided by the Laboratoire de santé publique du Québec (LSPQ), the Swiss National Reference Laboratory for *S. pneumoniae* and the Centre de recherche en Infectiologie de l’Université Laval strain collection.

Clinical samples were obtained from blood of children with pneumococcal bacteremia, from CSF of children with pneumococcal meningitis, in 2007-2009 in Cameroon [35], and from naso-pharyngeal swabs of children with pneumonia, in 2008-2010 in Switzerland. All samples were collected with the approval of the medical ethics committee of the Hôpitaux Universitaires de Genève. Written informed consent was obtained from parents.

### Bacterial culture and DNA extraction

DNA was extracted from clinical samples using the Purelink viral RNA/DNA minikit (Invitrogen, Burlington, ON, Canada). Reference bacterial strains were grown in the appropriate medium until a turbidity of 0.5 McFarland was obtained. Bacteria were centrifuged and resuspended in 5X TE buffer. DNA was extracted using the BD GeneOhm™ Lysis Kit (BD GeneOhm, San Diego, CA). Purified DNA was quantified on agarose gel and diluted to a concentration of 100 ng/µl and then to a concentration of 1 ng/µl. Genomic DNA was kept at 4°C.

### Primers and probes

The composition of the multiplex PCR primer mix is described in Table 1. Primer should be pooled in 1X TE to obtain final multiplex primer mix concentration as described in Table 1. Primers used for primer extension were composed of a proprietary tag sequence, specific for the INFINITI system, followed by a specific detection sequence. The tag sequence must be selected based on the system on which the assay is implemented and should be provided by manufacturers. The composition of the primer extension primer mix is listed in Table 2. However, Table 2 contains only the specific detection sequence of the primers. Each sequence must then be coupled to a tag sequence appropriate for the technology used for detection. Some primers should use the same tag sequence, as indicated by their TagID. The primer concentration in the primer extension mix is 500 nM for each primer, in 1X TE. Concentration of each primer in the primer extension solution is 50 nM.

**Table 1:** Genes targeted by the assay and composition of the multiplex PCR primer mix.

**Target gene**	**ProbeSet**	**Amplicon length**	**PrimerID**	**Sequence**	**Multiplex Primer Mix (µM**)	**Final concentration (µM**)
Integral membrane regulatory protein (wzg)	B, E	417 nt	SpneumoF004	GATCGATTTGTTGGGTGGGGTAGATG	1	0.05
			SpneumoF005	TGATTGATTTGTTGGGTGGAATTGATGT	1	0.05
			SpneumoF009	GATTGACTTATTGGGAGGGRTAGATGTTC	2	0.1
			SpneumoR006	CACATAGAGGTTACTGTCTGGCATTGC	1	0.05
			SpneumoR007	CAYATAGAGGTTACTATCTGGCATCGC	1	0.05
			SpneumoR008	CATCACATAGAGGTTACTGTCTGGTATTGC	1	0.05
Glycosyl transferase	I	199 nt	SpneumoF021	GTTTCGTTTACAACAATTCCAAGCCGTC	1	0.05
			SpneumoR022	GAGCGAAGATTTTCGTCCACATAGAC	1	0.05
Tyrosine-protein kinase (wze)	J	161 nt	SpneumoF023	GAGCCTTGGGATAAGTACATCTCTGT	1	0.05
			SpneumoF024	GAACCTTGGGACAAGTATATCTCTGT	1	0.05
			SpneumoR025	AACTTGRAAGCCATTAGCTACGC	1	0.05
Protein-tyrosine phosphatase (wzh)	C,D,F	617 nt	SpneumoF010	TTTGATGTAGATGAYGGTCCCAAGTCAA	2	0.1
			SpneumoR011	CATATGCTTCTGCCATATGRGGAGGTCT	2	0.1
			SpneumoR012	CATATGCCTGTTGCATATATGGAGGTCT	1	0.05
UDP-galactopyranose mutase (glf)	M	269 nt	SpneumoF033	GCTCATATCTTYCATACTTCTGATAAGGAGA	1	0.05
			SpneumoR032	AGATGTCTGTASCTACAAGAGAAATCGC	1	0.05
dTDP-d-glucose -dehydratase	P	176 nt	SpneumoF039	GGAGCTGGCTTTATCGGTTC	1	0.05
			SpneumoR040	GTCTACCAACTCCGCATCAGCAATG	1	0.05
Acetyl transferase	Q	333 nt	SpneumoF041	AGTTTGGGCAACATTTAGTGTGATTGCT	1	0.05
			SpneumoR042	TGTTGCTAACGAGCCAATTAGAGC	1	0.05
Capsular polysaccharide biosynthesis protein (wzd)	N et R	293 nt	SpneumoF043	CCTGAGTTTACTAGTATGACTCGG	0.5	0.025
			SpneumoF045	CCARAATATACGAGTACCACGCGA	1	0.05
			SpneumoF046	CCTGAGTTTACTAGTACGACTCGG	1	0.05
			SpneumoR036	TCTCAAAGAGTTAGCGATACGRCT	1	0.05
			SpneumoF047	AGCCAGAATATACAAGYACCACGCGTA	1	0.05
			SpneumoR048	TCGTAGAGAATTAGCGATACGACTG	1	0.05
			SpneumoR049	TCGTAKAGAATTAGCAATGCGACTG	1	0.05
Autolysin	*lytA*	201 nt	SpneumoF037	CCTCAAGTYGGCGTGCAAC	1	0.05
			SpneumoF038	CATTCCAACCRCCCCCAAC	1	0.05
Pneumolysin	*ply*	300 nt	SpneumoF014	GAGGGTAATCAGCTACCCGATGAG	1	0.05
			SpneumoR016	GAAAGCTATCGCTACTTGCCAAACCAG	1	0.05
M13 Internal Control	IC	167 nt	M13-IC3-F	GGAATGAAACTTCCAGACACCGTAC	1	0.05
			M13-IC3-R	ACCGGAAGCAAACTCCAACAGG	1	0.05

**Table 2:** Composition of primer extension multiplex.

OligoID	Sequence (Without tag assignation)	Probeset	TagID
SpneumoD003-B	CAATCAACAAAAGGTGATTGCGGCC	B	24
SpneumoD004-B	CAAYCAACAAAAGGTGATTGTGGCT	B	25
SpneumoD005-B	GCAACCAACAAAAGGTCATTGTAGCA	B	26
SpneumoD014-C	CATGTCCTCAAAYCCAAACTTTTTGGC	C	11
SpneumoD016-C	CTGATTGATATGGGRTGCTATACTCAGA	C	14
SpneumoD019b-C	CACATGTCCTCAAACCAAAACTTTTTGGAG	C	12
SpneumoD013-D	TTCGGGAAATAGCTAAGGAAGTSGCG	D	10
SpneumoD020-D	TTCGGGAAATTGCAAAAGAAGTAGCA	D	36
SpneumoD006-E	GGGAAGTTCCATTTCCCAGTTGGA	E	27
SpneumoD021-E	GGAAGTTCCATTTCCCAGTAGGG	E	32
SpneumoD022-E	AATGGAAAGTATTAYCCTGCAGGCA	E	33
SpneumoD009-F	ACAATGGTTCKCACCCCCTGC	F	6
SpneumoD023-F	AATGGTTCGCACCCCCTGT	F	37
SpneumoD024-F	AGACACAATCATTCGAACTCCTTGA	F	30
SpneumoD025-F	AGAGACAATGATTCGTACCCCTTGC	F	34
SpneumoD034-I	CTAGGATACTCTCCAAATTAATACCTTCAAATTC	I	7
SpneumoD035-I	CTAGGATACTCTCCAAATTAATACCTTCAAATTTA	I	8
SpneumoD036-J	CGAGGAACAAAGATTCCCTTACTC	J	9
SpneumoD037-J	GAGGAACAAAGATTCCCTTGCTT	J	13
SpneumoD042-M	CGTYACAACTCCCCAGAGTTTATTGAAG	M	19
SpneumoD043-M	GCGTTACAACTCCCCARAGTTTATTGAAA	M	20
SpneumoD044-N	TCGCAGGATGTTTTGGAGGAAGTTA	N	21
SpneumoD045-N	CGCAGGATGTTTTGGAGGAAGTTG	N	22
SpneumoD057-N	TCGCARGATGTATTGGAAAAGGTAGCG	N	15
SpneumoD058-N	TCGCAGRATGTATTGGAAAAAGTAGCG	N	15
SpneumoD059-N	TCGCAGGATGCATTGGAAAAAGTAGCG	N	15
SpneumoD047-P	TGTCCATTATGTTTACGAGAACTTTCCAGA	P	1
SpneumoD048-P	GTCCATTATGTTTACGAGAACTTTCCAGG	P	2
SpneumoD049-Q	GCTCGATTTGCTGTACCTTTATTTTTCATGATTTCATT	Q	3
SpneumoD050-Q	CGATTTGCTGTACCTTTATTTTTCATGATTTCAGG	Q	4
SpneumoD051-R	CTCGGATTTATGTAGTTAACCGTGG	R	5
SpneumoD052-R	GACTCGGATTTATGTAGTTARCCGTGA	R	29
SpneumoD053-R	CGACTCGGATTTATGTAGTTAACCGTA	R	31
SpneumoD054-R	CGCGAATTTACGTAGTGAATCGC	R	35
SpneumoD055-R	CCACGCGTATTTACGTAGTCAACCGT	R	16
SpneumoD056-R	CCACGCGAATTTACGTAGTGAATCAC	R	18
SpneumoD046	CACCATTATCAACAGGTCCTACCTGC	*lytA*	28
SpneumoD026	GCTACCAACGACAGTCGCCTC	*ply*	17
M13-IC3-D	TGTTGAGCTACAGCACCAGATTCAGC	IC	23

#### Microarray assay

Summary of the assay procedure is shown in Figure 1. Multiplex PCR was performed in a T1plus thermocycler (Biometra, Montreal Biotech, Montreal, Canada). Multiplex PCR was performed for each sample. The amplification solution was composed of 1X Platinum Taq buffer, 0.2 µM dNTPs, 1.5 mM MgCl2, 1µl multiplex PCR primer mix, 0.5 units of Platinum Taq DNA polymerase (Invitrogen, Burlington, ON, Canada), and 2.5 µl of cDNA, in a final volume of 20 µl. Prior to PCR, 10^7^ copies of phage M13amp18 RF1 DNA (New England Biolabs, Pickering, ON, Canada) was added to each reaction as an internal control. The PCR program consisted in the following steps: 60 s at 94°C followed by 39 cycles of 30 s at 94°C, 30 s at 55°C and 90 s at 72°C. Finally, the reaction was incubated at 72°C for 3 min. Then, 3 units of shrimp alkaline phosphatase (Clontech, Mountain View, CA), 7.5 units of exonuclease (Clontech) and 0.25 µl of 50X titanium DNA polymerase (Clontech) were added to the solution, which was incubated at 37°C for 20 min and at 94°C for 10 min. This step allows for the degradation of remaining dNTPs and PCR primers that were not used in the multiplex PCR. The subsequent steps were automated by the INFINITI analyzer (AutoGenomics Inc., Carlsbad, CA). The primer extension solution was composed of 1X Platinum Taq buffer, 2.5µM MgCl2, 156 µM d(A/G/T)TP, 10µM Cy5-dCTP, 2µl primer extension primer mix completed with water to 20µl per reaction. A volume of 20µl of primer extension solution was added to each reaction. Primer extension reaction consisted in the following steps: 60 s at 94°C followed by 39 cycles of 15 s at 94°C and 15 s at 50°C. Primer extension was done in the presence of Cy5-dCTP. Following the primer extension reaction, 80 µl of hybridization solution was added to each reaction. The total volume of 120 µl was then hybridized to a DNA microarray for 90 min at 42°C with high humidity. The tags on the extension primers hybridized to corresponding probes on the microarray. After hybridization, each chip was washed 5 times with 300 µl of 1X saline sodium citrate (SSC). Chips were dried and scanned using a confocal scanner. Microarray results have been submitted to Gene Expression Omnibus database (GSE45865).

### Result analysis

The algorithm used for *S. pneumoniae* detection and serotyping result analysis is summarized in Figure 2. Microarray results were controlled for background and negative control. For each probe a ratio between the mean of the background corrected signal of triplicate probes and background fluorescence was calculated. Positivity of the internal control probes (ratio > 3) confirmed the test validity for this sample. Sample was deemed positive for *S. pneumoniae* if the ratio of either the pneumolysin (*ply*) or the autolysin (*lytA*) probes was greater than 1.5. If the sample was positive for *S. pneumoniae*, the serotyping probes were analyzed to provide a serotype. Twelve positions of the capsular operon were typed at the nucleotide level. For each genotyped position, probes with ratios greater than the mean ratio for the sample were considered positive. If one or more probe was positive, the probe with the highest signal to background ratio was considered positive for the position. Each sample was compared to expected results for 92 serotypes and a score was calculated for each : (concordant probes - false positive) / (expected probes for serotype + discordant probes + false negative probes). Afterwards, the score for all serotypes were compared and, if the highest score obtained was higher than 0.4, the sample was associated to this serotype. If many serotypes shared the highest score, the sample was associated to this group of serotypes. Serotype determination software "Pneumotyper" and serotype database are available on GitHub (https://github.com/fredericraymond/Pneumotyper).

## Results

### Design of the assay

**Figure 3:**
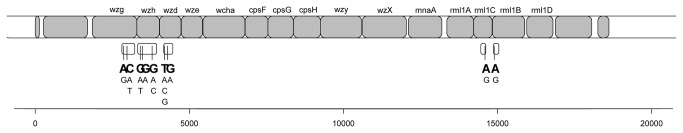
Capsular operon of *S. pneumoniae* serotype 19A. Expected amplicons are shown under the sequence, with genotyped positions marked with vertical lines. The expected genotype for serotype 19A is in large characters while the other possible genotypes are shown in smaller type. Positions are in nucleotides.

A machine learning approach was used to identify genes and single nucleotide variations within the capsular locus that, in combination, allow for the serotyping of *S. pneumoniae*. Coding sequences (CDS) were extracted from 90 capsular polysaccharide biosynthetic clusters of *S. pneumoniae* [28] and CDS were compared in order to form subgroups. Initially, the optimal combination of CDS necessary to identify the 13 vaccine serotypes of interest was determined using the Id3 decision tree algorithm implemented in the Weka software [33]. Selected CDS were mined to find polymorphisms specific to different capsular serotypes. Selected polymorphisms were added to the decision tree optimization parameters and targets were again optimized with Id3 to identify additional serotypes. Several iterations of this process were performed to increase the number of serotypes detected by the assay using a minimal number of targets. Detection primers were designed to identify these polymorphisms, which, in combination, permit serotype identification. Figure 3 provides an example of the nucleotides typed for serotype 19A.

### Evaluation with previously serotyped strains

The assay was evaluated on a collection of 166 cultures of *S. pneumoniae* previously serotyped by the Quellung assay, representing 63 different serotypes. Detailed results are shown in Table 3. All strains were found to be positive for *S. pneumoniae*, with all 166 strains positive for the pneumolysin gene (100%) and 165 positive for autolysin (99.4%). The strain negative for autolysin was of serotype 48 and was correctly serotyped. Serotyping using the assay allowed the correct identification of 126 (75.9%) samples and the misidentification of 14 (8.4%) samples to closely related serotypes of the same serogroup. These erroneous identification were serotype 6A identified as 6B (6/14), serotype 19B (2/14) or 19C (2/14) identified as serotype 19A, serotype 6B (1/14) identified as 6A/6C, serotype 24F (2/14) identified as 7C/24B/45 and serotype 28F (1/14) identified as 28A. Only 8 samples (4.8%) had erroneous serotyping out of the expected serogroup, and none accounted for misidentified vaccinal serotypes. They were of serotype 21, 41F, 42, 44, 45, and 46. Finally, 18 samples (10.8%) were positive for pneumolysin and autolysin but could not be serotyped because their serotyping score was lower than 0.4. The serotype yielded by samples with scores lower than the threshold was investigated. Of these, 8 would have been correctly serotyped and 10 would have been discordant if called positive. The latter were of serotypes 4, 10A, 10B, 15F, 39 and 43. Negative and discordant samples were retested and yielded similar results.

**Table 3:** Serotyping of 166 cultures of *S. pneumoniae* previously typed by the Quellung assay.

**Serotype as dermined by Quellung**	**Expected results**	**Vaccine**	**n**	**Genotyping concordant with Quellung**	**Genotyping yield appropriate serogroup**	**Genotyping results discordant with Quellung**	**Genotyping unable to serotype sample**
4	4	7	7	6	0	0	1
6B	6B	7	8	7	1 (6A or 6D)	0	0
9V	9A;9V*	7	7	7	0	0	0
14	5;14*	7	8	7	0	0	1
18C	18C	7	5	5	0	0	0
19F	19F	7	7	6	0	0	1
23F	23F	7	8	8	0	0	0
1	1	13	7	7	0	0	0
3	3	13	7	5	0	0	2
5	5;14*	13	6	6	0	0	0
6A	6A	13	10	3	6 (6B)	0	1
7F	7F	13	6	6	0	0	0
19A	19A	13	6	6	0	0	0
2	2	Non-Vaccine	1	1	0	0	0
6C	6C	Non-Vaccine	1	1	0	0	0
7A	7A	Non-Vaccine	1	1	0	0	0
8	8	Non-Vaccine	3	3	0	0	0
9N	9L;9N;13;20*	Non-Vaccine	3	3	0	0	0
10A	10A	Non-Vaccine	2	0	0	0	2
10B	10B	Non-Vaccine	2	0	0	0	2
10F	10C;10F;11A;11D;38*	Non-Vaccine	2	2	0	0	0
11A	10C;10F;11A;11D;38*	Non-Vaccine	1	1	0	0	0
12A	12A;12B;12F;46*	Non-Vaccine	1	1	0	0	0
12F	12A;12B;12F;46*	Non-Vaccine	6	6	0	0	0
13	9L;9N;13;20*	Non-Vaccine	1	1	0	0	1
15A	15A	Non-Vaccine	1	0	0	0	1
15B	15B;15C*	Non-Vaccine	1	1	0	0	0
15C	15B;15C*	Non-Vaccine	1	1	0	0	0
15F	15F	Non-Vaccine	1	0	0	0	1
16F	16F	Non-Vaccine	1	1	0	0	0
17F	17F	Non-Vaccine	1	1	0	0	0
18F	18F	Non-Vaccine	1	1	0	0	0
19B	19B	Non-Vaccine	2	0	2 (19A)	0	0
19C	19C	Non-Vaccine	2	0	2 (19A)	0	0
20	9L;9N;13;20*	Non-Vaccine	2	2	0	0	0
21	21	Non-Vaccine	2	0	0	2 (12A;12B;33B;33D;46)	0
22A	22A;22F*	Non-Vaccine	1	1	0	0	0
22F	22A;22F*	Non-Vaccine	1	1	0	0	0
23A	23A	Non-Vaccine	1	1	0	0	0
23B	23B	Non-Vaccine	1	1	0	0	0
24F	24F	Non-Vaccine	2	0	2 (7C;24B;45)	0	0
27	25F;27;31*	Non-Vaccine	1	1	0	0	0
28F	16A;28F*	Non-Vaccine	1	0	1 (28A)	0	0
29	29;35B*	Non-Vaccine	2	2	0	0	0
31	25F;27;31*	Non-Vaccine	1	1	0	0	0
32A	32A;32F*	Non-Vaccine	1	1	0	0	0
33F	33A;33C;33F;35A;35C*	Non-Vaccine	1	1	0	0	0
34	34	Non-Vaccine	1	1	0	0	0
35A	33A;33C;33F;35A;35C*	Non-Vaccine	1	1	0	0	0
35B	29;35B*	Non-Vaccine	1	1	0	0	0
35F	35F;47F*	Non-Vaccine	1	1	0	0	0
36	36	Non-Vaccine	1	1	0	0	0
37	37;47A*	Non-Vaccine	1	1	0	0	0
38	Untypable	Non-Vaccine	2	0	0	0	2
39	39	Non-Vaccine	1	0	0	0	1
40	40	Non-Vaccine	1	1	0	0	0
41F	41F	Non-Vaccine	1	0	0	1 (17F)	0
42	42	Non-Vaccine	1	0	0	1 (33A;33C;33F;35A;35C)	0
43	43	Non-Vaccine	1	0	0	0	1
44	11F;12F;44*	Non-Vaccine	1	0	0	1 (12A;12B;12F;46)	0
45	45	Non-Vaccine	2	0	0	2 (22A;22F)	0
46	12A;12B;12F;46*	Non-Vaccine	2	1	0	1 (21;28A;32A;32F)	0
48	40;48*	Non-Vaccine	1	1	0	0	0

* Serotype is assigned to a subgroup of probable serotypes

### Cross-reactivity with other bacteria species

In order to ensure its specificity, the assay was performed on twenty-four species of bacteria that could potentially be present in a clinical specimen, including ten *Streptococcus* species (Table 4). All species were positive for the internal control and negative for pneumolysin, autolysin and serotyping, except for *S. pseudopneumoniae*. As expected, *S. pseudopneumoniae* (BAA-960) yielded positive results for pneumolysin, autolysin and *wzd*. This strain of *S. pseudopneumoniae* has already been shown to contain these virulence genes [34].

**Table 4:** Cross-reactivity with common bacteria species.

**Species**	**Strain**	**Pneumolysin**	**Autolysin**	**Serotyping**	**Internal Control**
*Bordetella* *pertusis*	ATCC 9797	-	-	-	+
*Corynebacter* *jeikeium*	ATCC 15978	-	-	-	+
*Corynebacterium* *minutissimum*	ATCC 23348	-	-	-	+
*Corynebacterium* *pseudodiphteriticum*	ATCC 10700	-	-	-	+
*Corynebacterium* *xerosis*	ATCC 373	-	-	-	+
*Enteroccus* *faecalis*	ATCC 19433	-	-	-	+
*Escherichia coli*	ATCC 43886	-	-	-	+
*Haemophilus* *influenza*	ATCC 9006	-	-	-	+
*Klebsiella pneumoniae*	ATCC 27736	-	-	-	+
*Neisseria* *meningitidis*	ATCC 13077	-	-	-	+
*Pseudomonas aeruginosa*	ATCC 35554	-	-	-	+
*Rhodococcus* *equi*	ATCC 6939	-	-	-	+
*Staphylococcus aureus*	ATCC 29213	-	-	-	+
*Staphylococcus epidermidis*	ATCC 35984	-	-	-	+
*Streptoccocusmitis*	ATCC 49456	-	-	-	+
*Streptoccus* *pyogenes*	ATCC 12384	-	-	-	+
*Streptococcus* *cristatus*	ATCC 51100	-	-	-	+
*Streptococcus gordonii*	ATCC 10558	-	-	-	+
*Streptococcus oralis*	ATCC 10557	-	-	-	+
*Streptococcus parasanguinis*	ATCC 15912	-	-	-	+
*Streptococcus pseudopneumoniae*	BAA-960	+	+	-	+
*Streptococcus salivarius*	ATCC 7073	-	-	-	+
*Streptococcus* *sanguinis*	ATCC 10556	-	-	-	+
*Streptococcus* *vestibularis*	ATCC 49124	-	-	-	+

### Limit of detection for *S. pneumoniae* detection and serotyping

Limit of detection studies were performed on cloned pneumolysin and autolysin genes and on quantified genomic DNA of serotypes 9V and 23F. Plasmids were serially diluted and dilutions were tested with the assay. Pneumolysin could be detected at a dilution lower than 10 copies per reaction and autolysin was detected at a 100 copies dilution. Genomic DNA from laboratory strains were dosed on agarose gel and serially diluted in order to determine the sensitivity of serotyping using the assay. The 100 genome equivalent limit of detection for serotyping was consistent between all samples. Limits of detection lower than 100 and 500 genome equivalent were observed for pneumolysin and autolysin, respectively.

### Clinical samples

In order to validate that clinical specimens were suitable for serotyping using the assay, the test was performed on two sets of specimens obtained from patients, a series of 43 cerebrospinal fluid (CSF) samples [35], and a series of 59 nasopharyngeal aspirates (NPA). None of these samples have been serotyped with the Quellung assay. *S. pneumoniae* detection and serotyping was possible for the two types of specimens, demonstrating that culture is not required for serotyping using our method.

The 43 CSF samples were collected in Cameroon from patients with meningitis [35] and were previously tested positive for pneumolysin-specific real-time PCR as described by Corless and collaborators [36]. Using our assay, 40 of these specimens were positive for pneumolysin (93.0%), 34 were positive for autolysin (79.0%) and 35 were serotyped (81.4%). Serotype distribution of the 43 CSF samples from Cameroon, serotyped using the microarray assay described herein, was published by Gervaix and collaborators [35]. . The 59 NPA were obtained in Geneva, Switzerland, from children diagnosed with community-acquired and radiographically confirmed pneumonia of unknown etiology. Of these 59 specimens, 45 were positive for pneumolysin (76.3%), 34 were positive for autolysin (57.6%) and 41 were serotyped (69.5%), as shown in Table 5. These results demonstrate that pneumococcal culture is not required to conduct serotyping using the assay described herein.

**Table 5:** Distribution of serotypes detected in nasopharyngeal aspirates.

**Serotype**	**Positive NPA**
6A	4
6B	1
7F	3
8	1
9A or 9V*	1
9L, 9N or 20	4
10B or 34*	1
10C, 10F, 11A or 11D	2
14	2
16F, 23B, 27, 28A, 31 or 41A*	1
16F, 27, 28A, 31 or 41A*	3
16F, 28A, 31 or 41A*	1
17F	1
19A	6
19B	1
19C, 22A or 22f*	1
19F	1
19F or 23A*	1
23A	1
24A	1
39	1
43	1
48	1
27 or 31	1
*S. pneumoniae* detection only	6
Negative	12

* Subgroup due to limited sample concentration

## Discussion

Accurate serotype determination of *Streptococcus pneumoniae* is of paramount importance to assess the impacts of pneumococcal vaccines and to monitor emergent serotypes. In order to simplify *S. pneumoniae* serotyping, we created a test that relies on a combination of genomic properties to identify serotypes. In this study, we validated the identification of 46 serotypes, 22 of which were precisely identified and 24 assigned to a subgroup of probable serotypes (Table 3). This is done by genotyping only 12 positions in the capsular operon. Serotypes included in the 13-valent vaccine were correctly assigned to serotypes, although serotypes 6A and 6B were sometimes not resolved. Bioinformatics analyses of available capsular operon sequences suggest that several other serotypes could be identified using this assay, but they have not been validated in the current study. In such cases, and for vaccinal serotypes 6A, 6B and 19A, it would be appropriate to confirm serotype determination using another method, such as the Quellung assay. Validation could also be performed to increase the precision of serotyping when the assay provides a subgroup of serotypes or unvalidated serotypes. We also tested the assay on clinical specimens consisting in 56 nasopharyngeal aspirates from patients with pneumonia and 43 cerebrospinal fluid samples from patient with pneumococcal meningitis, demonstrating that the assay do not require culture prior to serotyping.

The assay targets 12 polymorphic positions located in genes of the capsular operon. It was designed with sufficient redundancy to allow for the occasional false negative probe so that, in most cases, missing probes will not prevent serotyping. If several variants are possible at a single position in the known *S. pneumoniae* population, all polymorphisms are accounted for in the design. This provides redundancy that may counterbalance missing results, making the assay more robust. This is especially useful in specimens with low sample concentration or in specimens for which capsular gene sequences may differ from reference sequences. In some cases, a precise identification of serotypes is theoretically possible, but missing signal for some polymorphisms prevent precise serotype identification. Thus, the results for these samples are a group of serotypes. This could be caused by sequence variations in target genes and could be resolved by testing the samples using another method.

The assay was validated on 166 cultures of *Streptococcus pneumoniae* covering 63 different serotypes. Fourteen samples were not identified as the appropriate serotype but were correctly assigned as members of the correct serogroup. Six samples of serotype 6A were identified as serotype 6B and one serotype 6B was identified as 6A/6C. It is implicit in the literature that serotypes 6A and 6B are difficult to discriminate using DNA-based assays. Indeed, the vast majority of previously published nucleic acid serotyping methods do not discriminate serotype 6A from 6B [6,14–22,27,32]. Moreover, 6A pneumococci with a *wzh* sequence previously observed in 6B serotypes have been reported in the Netherlands, highlighting the genetic diversity of this serogroup [26]. Assay designs based on phylogeny can provide relevant information, but can prove unreliable in some cases, such as in distinguishing between serotypes 6A and 6B. Designs based on functional polymorphisms, such as functional *wciP* genetic differences, could be more robust and reliable for these serotypes [29]. Serotypes 19B and 19C have been misidentified as serotype 19A. This is unexpected since the profile observed for these samples is not concordant with the published sequences for these serotypes [28]. It is of note that most molecular assays do not identify serotypes 19B and 19C, and that only a handful of molecular assays have been validated with serotypes 19B or 19C [15,22,32]. Variant 19F capsular genes have also been reported, leading to false positive 19A identification using PCR [37]. This highlights one of the challenges involved in validating *S. pneumoniae* serotyping assays, since it is difficult to obtain several representative strains of all serotypes. Using our assay, serotypes 24F was serotyped as 24B and 28F as 28A. Similar results were obtained with sequetyping [27]. The other misidentified samples were of low prevalence serotypes. Discordant samples outside of the serogroup were of serotypes 21, 41F, 42, 44, 45. Negative serotyping was obtained for 18 samples, 8 of which would have been correctly serotyped if their score had not been under the threshold of 0.4. They include 2 samples of serotype 3, and one sample for each of the following serotypes : 6A, 6B, 13, 14, 15A and 19F. These false negatives could be due to sequence variations that affect either PCR amplification, leading to low signal for some genotyping probes, or to mutations affecting the nucleotides that are genotyped by the assay, leading to missing signal or genotyping that do not conform with the information available in the serotyping database. This highlights the main problems that can be observed when a limited number of reference sequences are available to design assays.

In a recent study, Scott and collaborators used a glass-based microarray to detect the presence or absence of capsular genes, the combination of which allowed the identification of serotypes [31]. Instead of targeting a large number of capsular genes, the method described herein relies on the genotyping of selected nucleotides to identify serotypes. The use of a limited number of targets allowed the development of a multiplex PCR, which improves the limit of detection of the assay and eliminate the need for culture.

An advantage of this assay is that raw data from experiments can be reanalyzed upon the addition of new entries to the serotyping database or modifications to the identification software. Notably, new DNA sequences for which the serotype is known can help validate the assay and improve its robustness. The sequencing of additional *S. pneumoniae* capsular operons for relevant serotypes could improve the specificity of this assay. By testing the assay on several *S. pneumoniae* strains of the same serotype, we observed divergences between the expected genotype based on already determined capsular sequences and the actual results. For example, serotypes 19B and 19C produced the same genotyping profile as 19A, while the previously determined sequences hinted to a different profile. The same was observed with 6A compared to 6B. This suggests that actual strain-to-strain sequence variability within a single serotype could impact on the results of not only this assay, but on other molecular assays too. Thus, the sequencing of more isolates of each serotype would vastly improve serotyping assays that rely on genome sequences. In the future, new serotypes could be added to the analysis software provided that their capsular genes have been sequenced. The bioinformatics approach used to create this assay could also be applied to a larger sequence dataset, which should include the genome of several strains of each serotype, in order to create an improved molecular serotyping assay. A second multiplex PCR could be devised to genotype supplementary positions and thus improve the specificity and serotype coverage of the assay. In the end, as with other serotyping assays based on PCR and genotyping, immunological methods, such as the Quellung assay, may be required to confirm serotypes [6,37].

To facilitate the use of this assay in a clinical setting and to perform large scale epidemiological studies, we adapted the assay for use in the AutoGenomics INFINITI analyzer, an automated molecular diagnostic system that both reduces the hands-on time and the number of steps required to perform the assay. Twenty-four samples can be processed simultaneously with an overall processing time of 16 hours, with less than one hour of hands-on time. This system has been successfully used for the diagnostic of multiple respiratory viruses [38]. The serotyping assay could be adapted for additional platforms, such as the Luminex (Millipore), the BeadExpress (Illumina), or amplicon sequencing.

As exemplified by our Cameroon study [35] and several others [39], the serotypes circulating in developing countries are different from those observed in developed countries. This stresses the need for appropriate monitoring of vaccinal strains for surveillance. In order to properly describe the serotypes found in a country where no previous serotype prevalence studies have been performed, a test that allows the identification of a great number of serotypes would be beneficial. An assay such as the one described herein constitutes a reliable tool for a first screening of the serotypes circulating in unstudied populations. An approach based on polymorphism detection instead of serotype-by-serotype targets would allow a more versatile first screening of previously unstudied regions, where a wide coverage of serotypes is required.

## Conclusions

This microarray-based assay uses a single test to identify most *S. pneumoniae* serotypes or serogroups. It is therefore useful for many types of infections, sample sources and for different countries. The current embodiment of the assay constitutes at this time a reasonable compromise between complexity and yield. Basically, it is a proof of concept that *S. pneumoniae* serotype identification can be performed by typing a limited combination of carefully selected polymorphic positions. Indeed, 22 serotypes can be precisely identified and 24 can be assigned to a subgroup of serotypes by genotyping only 12 nucleotides of the capsular operon. While the assay could be improved by the addition of new targets, the concept behind the assay could be used to devise methods to solve problems of similar complexity.
